# What Is My Role? A Qualitative Study of Labor, Birth, and Postpartum Experiences of Partners

**DOI:** 10.1111/nhs.70280

**Published:** 2025-12-28

**Authors:** Beatriz Pereda‐Goikoetxea, Montserrat Fiaño‐Santos, Sonia Tomas‐Riscado, Marijose Uranga‐Iturriotz, Joseba Xabier Huitzi‐Egilegor

**Affiliations:** ^1^ Faculty of Medicine and Nursing. Department of Nursing II University of the Basque Country (EHU) San Sebastián Gipuzkoa Spain; ^2^ Donostia University Hospital, Maternity Service San Sebastián Gipuzkoa Spain

**Keywords:** family‐centered care, fathers, midwifery, partner involvement, parturition, postpartum period, qualitative research

## Abstract

This study explores partners' roles during labor, birth, and the postpartum period, and the factors influencing their performance. A qualitative interpretive phenomenological approach was adopted to explore participants' lived experiences. A total of 31 partners participated in semi‐structured face‐to‐face interviews using open‐ended questions. The roles performed by partners can be grouped into a single role: comprehensive caregiver of the birthing woman and the newborn, encompassing physical, emotional, and social care. Facilitators included effective communication, access to information, a sense of involvement, and prior experience, whereas barriers comprised insufficient support, exclusion, uncertainty, and undervaluation of their role. This dynamic role evolves through sociocultural expectations, feedback with professionals, and institutional policies, highlighting the importance of supporting partners to strengthen family‐centred care.

## Key Points

- Partners (both women and men) fulfill a comprehensive caregiving role encompassing physical, emotional, and social domains. The social dimension includes acting as a spokesperson, serving as a liaison, and participating in decision‐making. This role is dynamic and continuously evolving, highlighting the importance of supporting partners to strengthen family‐centred care.
- This study identifies three distinct forms of decision‐making within the partner's social role: joint decision‐making, supportive decision‐making, and autonomous decision‐making. This nuanced categorization offers new insight into how partners participate in clinical and relational processes during labor, birth, and the postpartum period.
- The study highlights previously underexplored barriers to partner involvement, notably the undervaluation of partners' caregiving role and limited institutional support, underscoring the need for policies and practices that strengthen family‐centred care.

## 1 Introduction

For women, partner presence during childbirth is considered beneficial, as it increases confidence and enhances coping ability during labor (World Health Organization 2020a, 2020b). Moreover, such involvement contributes to psychological well‐being (Hoffmann et al. 2023), and is associated with a higher likelihood of vaginal birth, reducing the need for interventions such as caesarean section or operative vaginal delivery (Dubey et al. 2023). Furthermore, it significantly affects the partner's adaptation to the new role and promotes emotional bonding with the newborn (Michałek‐Kwiecień et al. 2022). This dynamic not only promotes the well‐being of family members (Watkins et al. 2024), but may also confer public‐health benefits (Wynter et al. 2021). Accordingly, partner presence is regarded as essential for a positive birth experience (Wanyenze et al. 2022), and the WHO recommends continuous support from a companion of the woman's choice during labor and birth (World Health Organization 2020a, 2020b).

Evolving perinatal care models have increasingly emphasized family‐centred approaches, which recognize the importance of integrating partners and close family members in caregiving (Bohren et al. 2017). Traditionally, care focused primarily on the woman, prioritizing her physical and emotional well‐being. In contrast, family‐centred models aim to provide a holistic understanding of the birthing woman within her family context, enhancing satisfaction with care and potentially improving perinatal outcomes (Wanyenze et al. 2022; Small et al. 2025). By actively involving partners, these models support parents in adapting to new roles and foster a mutually supportive environment (Wynter et al. 2021).

This study is informed by a family‐centred care framework, which emphasizes the interdependence of family members during childbirth. This perspective positions partner involvement as a key component of supportive perinatal care, recognizing its contribution to emotional well‐being, parental role development, and improved perinatal outcomes (Bohren et al. 2017; Wynter et al. 2021). Additionally, this study interprets partner involvement using Watson's Theory of Human Caring (Watson 2008), conceptualizing care as a transpersonal process that supports holistic well‐being. This framework, together with Watson's Theory of Human Caring, provides a basis for examining how partners participate and how their involvement is shaped by contextual factors.

Partner roles in childbirth have evolved markedly in recent years, especially with the promotion of family‐centred care models that respect the birth process. Longworth et al. (2021) found that partner roles range from passive positions (e.g., observers) to active participation in the birthing process. Reported partner support actions predominantly include provision of emotional support; meeting physical‐care needs; communication tasks (Wanyenze et al. 2022), psychological support (Vischer et al. 2020), physical assistance and protection (Wanyenze et al. 2022), advocacy for the birthing woman; caregiving tasks; information exchange and expression of parental wishes (Longworth et al. 2021), and participation in decision‐making (Ngai and Xiao 2021). Such participation may involve partners neglecting their own needs to prioritize the birthing woman's needs (Harrison et al. 2024; Uribe‐Torres et al. 2024).

Facilitating factors for partner participation in the perinatal process include prior birth experience (Mulugeta et al. 2024), prenatal parental education (Nambile Cumber et al. 2024; Palioura et al. 2023), particularly when offered free of charge and outside working hours (Wynter et al. 2021), midwifery training and support (Wynter et al. 2021), support for midwives (Schmitt et al. 2022; Wanyenze et al. 2022), and access to accurate information (Griffith et al. 2025; Nambile Cumber et al. 2024). Identified barriers to partner participation include resource allocation, organization of care, facility limitations, and cultural attitudes (Sun et al. 2025; Kabakian‐Khasholian and Portela 2017). Additional obstacles include insufficient consideration for partners; individual, social, cultural and service‐level characteristics (Wynter et al. 2021), poor communication (Harrison et al. 2024), restrictive gender norms (Watkins et al. 2024), and lack of recognition by healthcare professionals (Baldwin et al. 2018).

Several authors have described the partner's role during childbirth as ambiguous or confusing (Vahtel et al. 2021). Partners have reported feeling like ‘spare parts’ (Roberts and Spiby 2020) or mere visitors (Hodgson et al. 2021). This lack of role clarity reflects insufficient information or understanding and contributes to partners’ uncertainty about how to support the birthing woman effectively during labor (Elmir and Schmied 2022).

Despite increasing interest in partner roles during childbirth and the perinatal period, most studies have focused predominantly on fathers in heterosexual relationships, thereby overlooking diverse family configurations. More recently, research has begun to examine the experiences of same‐sex couples within maternity services (Denvir et al. 2025). This shift reflects the need for a more inclusive approach to family diversity, recognizing that both heterosexual and LGBTQ+ couples contribute to perinatal support (Fisher et al. 2021).

Accordingly, this study aims to explore the roles of partners during labor, birth, and the postpartum period, as well as the factors influencing their performance. Exploring these experiences can provide guidance for healthcare professionals on optimizing partner support and well‐being, with the aim of enriching the perinatal experience and promoting family well‐being.

## 2 Methods

### 2.1 Setting

The study was conducted at Donostia University Hospital in northern Spain. The hospital provides comprehensive community healthcare services to a population of approximately 360 000. Additionally, it functions as the public tertiary referral hospital for healthcare organizations across the province of Gipuzkoa, serving a catchment population of approximately 720 000. The number of births was 3053 in 2023 and 2981 in 2024.

In this population, 67.9% of mothers are aged 30–39 years, 24.7% are of foreign nationality, and 95.2% of births occur in public hospitals, while 4.0% take place in private centres and 0.8% occur at home. Furthermore, 50.6% of births are first‐time deliveries (Eustat 2025).

At Donostia University Hospital, childbirth care follows multidisciplinary protocols that prioritize safety, humane care, and family involvement (Osakidetza 2018a, 2018b). Although the hospital's approach encourages partner involvement to foster a supportive and emotionally safe environment during birth, the partner's exact role is not explicitly defined in institutional policy. This lack of formal definition may influence how partners engage in perinatal care and the experiences they report, highlighting the relevance of exploring their roles within the institutional context. In addition, a one‐to‐one care model is implemented, in which a midwife provides continuous, dedicated support to each birthing woman throughout labor and birth.

### 2.2 Study Design

The study employed a qualitative methodology using an interpretative phenomenological analysis (IPA) approach to obtain a detailed examination of personal lived experiences (Eatough and Smith 2017). Using this approach, participants’ experiences were described and interpreted, recognizing that full understanding requires consideration of both participants’ and researchers’ perspectives. This phenomenon is described by Smith et al. (2022) as the “process of double hermeneutics”.

### 2.3 Participants and Recruitment

Participants were the partners (both women and men) of birthing women. They were recruited by two midwives (M.F.‐S. and S.T.‐R.) between February and May 2023 in the postpartum unit of Donostia University Hospital, within 48 h after delivery, which corresponds to the typical hospital stay for uncomplicated births and ensured that all eligible partners could be approached before discharge. To minimize recruitment bias, midwives not involved in participants' clinical care approached eligible candidates in line with the study's inclusion criteria. Participants were selected using purposive sampling, and the sample size was determined by the principle of data saturation (Saunders et al. 2018). A total of 46 partners were initially approached. Of these, 15 ultimately did not participate: 9 withdrew their consent, 5 could not be reached by phone, and 1 did not attend the scheduled interview. Therefore, the final number of participating partners was 31.

Partners provided written informed consent to participate after receiving oral and written information about the study's purpose. Thereafter, participants completed a questionnaire collecting sociodemographic and obstetric characteristics (Table 1) and contact details. Inclusion criteria were: being the partner (regardless of gender) of the birthing woman and having been present during the perinatal process; adequate oral and written comprehension of Spanish and/or Basque; age ≥ 18 years; and capacity to understand and sign informed consent. Participation was restricted to partners of women who had delivered a liveborn infant at ≥ 37 weeks' gestation.

**TABLE 1:** Characteristics of partners and obstetric details of mothers and childbirths (*n* = 31).

Characteristics of partners	*N* = 31 *N* (%) or mean ± standard deviation
Age (years)	
Age range: 27–43	35.74 ± 4.015
Gender	
Male	30 (96.8%)
Female	1 (3.2%)
Country of birth	
Spain	29 (93.5%)
Other countries (Argentina, France)	2 (6.5%)
Place of residence	
Urban	10 (32.3%)
Rural	21 (67.7%)
Education level	
Lower education	1 (3.2%)
Higher education	30 (96.8%)
Employment status	
Employed	30 (96.8%)
Unemployed	1 (3.2%)
Parity	
Primiparous	11 (35.5%)
Multiparous	20 (64.5%)
Participation in antenatal preparation courses	
No	16 (51.6%)
Yes	15 (48.4%)
Characteristics of childbirth	
Gestational age at delivery (weeks)	
Range: 37–42	39.87 ± 1.312
Onset of labor	
Spontaneous onset	23 (74.2%)
Induced labor	8 (25.8%)
Mode of birth	
Spontaneous vaginal delivery	29 (93.5%)
Assisted vaginal delivery (instrumental)	2 (6.5%)
Analgesia/anesthesia used	
None	2 (6.5%)
Local anesthesia	3 (9.7%)
Epidural anesthesia	23 (74.1%)
Nitrous oxide	3 (9.7%)
Neonatal hospitalization	
No	26 (83.9%)
Yes	5 (16.1%)

Exclusion criteria were: twin pregnancies; caesarean‐section births; cases attended by the recruiting researchers; and situations in which the birthing woman or the newborn remained in a serious clinical condition after delivery.

### 2.4 Data Collection

At 8 weeks postpartum, the principal researcher (a midwife; B.P.‐G.) telephoned participants to arrange a semi‐structured, face‐to‐face interview at a time convenient for them. Interviews were conducted by the principal investigator between April and July 2023, using a thematic interview guide (Table 2).

**TABLE 2:** Thematic guide for the semi‐structured interview.

Topics	Purpose within IPA framework	Interview questions
Birth experience	Explore personal meaning‐making and emotional responses to the event	How would you describe your birth experience? How did you feel during labor and birth?
Priorities and needs	Understand perceived needs, values, and expectations	Which needs do you prioritize during labor, birth, and postpartum? What do you consider most important during labor and birth?
Expectations of role	Identify personal beliefs and assumptions	What were your expectations about your role during childbirth?
Participation	Explore enacted roles and behaviors	Did you wish to participate in labor, birth, and postpartum? If so, in what ways did you participate? Please describe.
Role in childbirth	Explore actual participation, functions performed, and personal meaning of the role	How was your participation? What functions or tasks did you perform?
Decision‐making	Understand perceived autonomy and involvement	How would you like to be involved in decision‐making during labor and birth?
Role meaning‐making	Deepen interpretation of actions and significance	What has the birth experience meant to you? What did your participation mean to you?
Closing question	Allow emergence of unanticipated topics	Is there anything else you would like to add?

The semi‐structured interview guide was designed following IPA recommendations for open, exploratory interviewing. Although the questions were concise, they served as broad prompts intended to elicit detailed narratives about participants' lived experiences. In accordance with IPA methodology, the questions were intentionally open and non‐directive, allowing participants to guide the direction and depth of the conversation, while the interviewer used follow‐up probes (e.g., “Can you tell me more about that?”, “How did you feel in that moment?”, “What did that mean for you?”) to encourage reflection. The guide covered the following thematic areas: birth experience, priorities and needs, expectations of role, participation, role in childbirth, decision‐making, role meaning‐making, and a closing question.

With participants' permission, interviews were audio‐recorded. Interviews were conducted in participants' homes or in cafeterias and had a mean duration of 37.26 min.

### 2.5 Data Analysis

**FIGURE 1:**
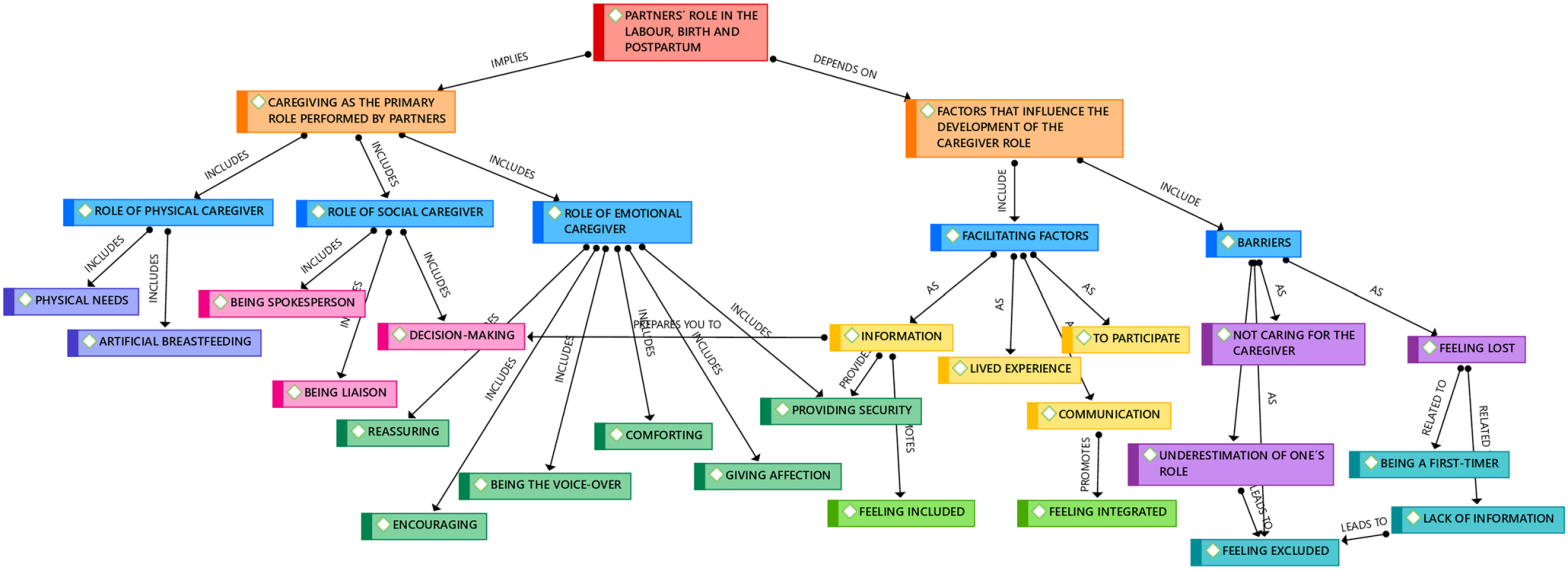
Creation of the semantic network in the analysis process.

Data were analyzed using Interpretative Phenomenological Analysis (IPA; Smith et al. 2022). Verbatim transcripts were read and re‐read independently by all researchers to perform initial coding. Subsequently, four researchers (B.P.‐G., M.F.‐S., S.T.‐R. and J.X.H.‐E.) compared codes to generate categories and potential themes, progressing from descriptive coding to interpretative analysis. Connections between emerging themes were analyzed and grouped to structure the analysis around central concepts. Subthemes were then identified using thematic maps (Figure 1).

Table 3 presents an example of this analytic process. The analytic process was iterative, with ongoing review and revision of the data. Emerging ideas were recorded in memos, accompanied by ongoing reflexive consideration of researchers' assumptions and preconceptions to mitigate subjective bias. Data saturation was monitored throughout the analysis process; saturation was considered reached when no new codes or themes emerged from subsequent interviews (Saunders et al. 2018). Discrepancies were discussed until consensus was reached among the research team. Atlas.ti version 8 (Friese 2017) was used to manage, sort, retrieve, and compare data during analysis.

**TABLE 3:** Description of the analysis process.

Examples of meaning units	Codes	Category	Subtheme	Theme
“The communication with the people around, because when the baby is born, the mother is focused on the baby and doesn't have her phone. So, the partner can inform, especially the parents who are not here…” (23:23).	Union communication outside family	Spokesperson	Role of social caregiver	Caregiving as the primary role performed by partners
“If the family is to be informed, I will also be the link with the outside” (10:15).				

The research team comprised three midwives and two nurses, all experienced in qualitative methods. No prior relationships were observed between researchers and participants, reducing potential bias and promoting trustworthiness. Reflexivity was maintained through continuous self‐reflection and memo writing, which facilitated the identification and critical examination of researchers' assumptions and specifically helped to address potential bias related to the lead investigator's dual role as a midwife. Regular team discussions, peer debriefing, and cross‐checking of codes supported consensus, validated themes, reduced bias, and increased rigor in data interpretation.

### 2.6 Research Ethics

The research ethics committee of the Gipuzkoa Health Area granted ethical approval for this study (BPG‐PHP‐2022‐01). Participation was voluntary, and both oral and written informed consent was obtained. Participants could withdraw from the study at any time without repercussions. Transcripts were anonymised and assigned pseudonymous codes to ensure participant confidentiality. Paper records were stored under lock and key, and digital data were held in a secure, password‐protected account.

### 2.7 Rigor

Rigor was addressed according to Lincoln and Guba (1985) criteria—credibility, transferability, confirmability, and dependability—which underpinned the trustworthiness of the findings. Credibility was enhanced through extensive verbatim quotations from interviews, which supported the study's interpretative claims. Transferability was supported by providing a detailed description of the study context and data‐collection procedures, and by assessing the representativeness of the dataset. Confirmability was promoted through reflexive practices, including the maintenance of a reflective diary to identify and minimize potential researcher bias. Dependability was reinforced through investigator triangulation and the use of qualitative analysis software to organize data systematically. The Consolidated Criteria for Reporting Qualitative Research (COREQ) guidelines (Tong et al. 2007) guided the reporting of this study, and the use of Interpretative Phenomenological Analysis (IPA) further strengthened the methodological rigor by enabling an in‐depth understanding of participants' lived experiences.

## 3 Results

The study included 31 partners, primarily fathers (96.8%), along with one female partner from a same‐sex couple. Most participants were born in Spain (93.5%), and employed (96.8%), with a mean age of 35.7 years. The majority lived in rural areas (67.7%) and held higher education degrees (96.8%). Regarding parity, 35.5% were first‐time parents, and 48.4% had attended antenatal preparation courses. Spontaneous vaginal births predominated (93.5%), with epidural analgesia used in 74.1% of cases (Table 1).

Two main themes emerged from the analysis: (1) caregiving as the primary role undertaken by partners, and (2) factors influencing the development of that role.

### 3.1 Theme 1: Caregiving as the Primary Role Performed by Partners

All participants identified caregiving as their principal role during labor, birth and the postpartum period. They emphasized that providing care required presence and active involvement. Their presence was not merely perceived as a duty but as a deliberate choice that strengthened their connection to the moment and to their partner.

I knew I had to be there, and at the end of the day, there was no better person than me to be there (10:22).

Being always present is how we will be able to experience it, otherwise we will end up being guests of honour, but we will not actively participate in this. It seems to me that participating is one of the best decisions I have ever made, and it has made me get through this much more intensely (25:73).

I can't imagine not being there; It's the first moment and I have got to be there. I can't imagine anything else; I can't be out there thinking about what could be happening. I just have got to be there for myself (29:55).

Additionally, partners described themselves as the most knowledgeable about the woman's needs and as her primary source of reassurance. This perception was often conveyed through small caring gestures that reflected emotional attunement.

It is very important to know how to read what is happening to the other person. Right now, I see she starts licking her fingers, Oh! Want some water? I'll bring it to you (7:46).

Three dimensions of the caregiver role were identified: physical, emotional, and social care. These dimensions are grounded in a holistic bio‐psycho‐social perspective of the person.

#### 3.1.1 Subtheme 1: Role of Physical Caregiver

Physical care targeted the bodily needs of the woman and the newborn, including providing drinking water, assisting with mobility, and supporting hygiene and grooming. Partners described this support through multiple concrete actions.

If she needs water or a towel, if she wants to shower, if she has to change the sanitary pads… meeting their needs is paramount (4:23).

Since this time she was going to try breastfeeding him, at that moment, you can't do anything, but you can take off the diapers, you can be with him (12:29).

I think in everything except breastfeeding. I participated in everything from personal care, personal hygiene, clothing… (20:62).

Bottle feeding (artificial breastfeeding) was identified as a means for partners to participate in newborn care; participants described it as promoting partner–newborn bonding and supporting the mother's recovery. Participants' accounts emphasized shared responsibility, emotional bonding with the newborn, and support for the mother's recovery as key benefits of bottle feeding.

So, we agreed that the best solution to be the two of us and actually be two in motherhood was the bottle, which has helped a lot, we guess (14:36).

I can help you much more if the milk is from formula. I think this is a two‐person matter. I have got to spend the nights like you do. It is not something that is exclusively the mother's responsibility, and I am able to help with this (17:47).

We share the responsibility for care equally; the three children have been bottle‐fed. From the moment the girl was born, I was there, also giving her the bottle at night while my wife was resting; this has helped us a lot. In the end her recovery has been good and quick, and I think it was largely for that reason. As for the bond I have had with the girl from the beginning seems to me to be like that of my wife, with the same involvement and workload (26:59).

#### 3.1.2 Subtheme 2: Role of Emotional Caregiver

Emotional care focused on providing support, offering comfort, showing affection, conveying security, reassuring, acting as a voice, and encouraging the woman. Partners helped the woman to orient herself in time and space during both labor and the postpartum period.

That voice‐over, you know, is just reassuring. Even though you're in a lot of pain and you're scared, you at least know that you have a partner who is going to be there with you and support you (3:43).

My role was above all to reassure her, so that she would not feel alone, and to stay beside her (9:31).

My participation was to be by her side, encourage her, reassure her, and help her in any way I could (15:25).

She does not remember many things, what happened, at what time. She told me: what happened here? She has erased that memory a little bit (29:54).

#### 3.1.3 Subtheme 3: Role of Social Caregiver

Social care aimed to manage the social environment through decision‐making, acting as a spokesperson and serving as a liaison. Decision‐making emerged as a particularly salient aspect of social care, encompassing diverse forms of involvement that reflect the partner's active role during the perinatal process.

Involvement in decision‐making was deliberate and manifested as: consensual decisions between partners, supportive decisions, and individual decisions. Consensual decisions were typically made before labor and were often recorded in a birth plan.

Indeed, it is up to both of us, and we both participate. This is the family we have created, and we both decide things for our children (11:26).

It seems to me that the partner or person who is going to help in the birth has to be aware of what they have talked about among themselves, in order to decide what to do at certain times (16:24).

Supportive decisions were related to interventions that directly affected the woman, such as the administration of epidural analgesia and choices about breastfeeding. In such situations, partners offered their opinion while supporting and respecting the woman's wishes.

I told her that it was a decision that directly affected her and that I would respect and support the decision she would make (23:60).

The issue of breastfeeding is something that I have left in her hands. I can't make the decision whether to breastfeed or bottle‐feed. <The decision you make will be the right one and I am here to support you> (25:67).

When required, the partner made decisions and communicated them to health professionals. Partners identified themselves as the least emotionally affected and the most capable of making unilateral decisions as the principal decision‐maker.

We were taking the time of contractions, now we have got to go to the hospital (12:24).

In this second birth I felt more empowered to, at certain moments, decide and make my decision known (29:43).

She told me that she wanted the epidural; I went to them and they asked me how she was doing. I said: <The epidural>. They went to talk to my wife and said, <Are we going to try a little?> and I told them: <No, no, we want the epidural; she's shattered> (30:20).

Whenever the woman was unable to communicate, partners served as spokespersons, voicing previously made decisions and any directives recorded in the couple's birth plan.

If she were focusing on childbirth, I would be the one caring for everything. It was necessary for the birth plan to work out well, to be sure that it followed what we both wanted (13:46).

The liaison role involved communicating with healthcare professionals, liaising with the woman when the newborn required hospitalization, and informing family members. Participants naturally assumed this role as part of their ongoing engagement throughout the perinatal process.

It was clear to me that my role was that, considering the expectations she had, she would not have to say I need this or that, but that I would handle the communication with the midwives (4:25).

You want to be in two places because the mother has just given birth in one place and the child is in another. At least I could tell her the news the paediatricians were giving us (6:2).

If the family is to be informed, I will also be the link with the outside (10:15).

### 3.2 Theme 2: Factors That Influence the Development of the Caregiver Role

#### 3.2.1 Subtheme 1: Facilitating Factors in the Development of the Caregiver Role

In the accounts of the partners, several factors were identified as facilitating the assumption of the caregiver role, including communication with professionals, access to information, a sense of involvement, and prior experience.

In their interactions with professionals, partners emphasized the importance of receiving personalized care, where trust and empathy created a sense of safety and tranquility. Additionally, the provision of information and clear guidelines supported their integration into the process.

It is good to be given some support, to be comforted, to be guided, to have things explained to you, to feel that you are in a safe environment. Above all, to feel supported (1:25).

Partners requested accurate and up‐to‐date information, delivered with consistent criteria across professionals, to enable active decision‐making, understanding of processes, and a greater sense of safety and reassurance. However, this information was sometimes recognized as being difficult to assimilate until experiencing it first hand, specifically that which was related to complex situations.

They should unify the criteria and update them, not just report on the beautiful things in the classes (14:71).

Being informed seems very important to me. One has to be aware of everything in order to be able to actively make decisions (16:23).

With respect to childbirth, you know that it is a unique, very special, very hard process, and that you have to endure it. You can mentally prepare yourself, but you are not aware of what you are going to experience. More information? Even if you are given all the information in the world, it is not the same as experiencing it; you have to take the bull by the horns; there is no other way (23:16).

Yes, it makes you feel more secure to be informed (28:22).

Public education classes were valued as a means to obtain information. However, a minority of partners did not attend, citing schedule incompatibility, lack of invitation from professionals, or childcare responsibilities. Other sources of information included social networks, books, and private preparations.

It was impossible considering my work schedule; I couldn't attend at any hour (21:16).

Active participation enhanced their sense of inclusion. In addition to providing care, partners repeatedly described practical tasks that contributed to their sense of inclusion during labor, birth, and the postpartum period. Examples included preparing the maternal health record, readying the car, organizing transport, arranging childcare for other children, collecting the hospital bag, timing contractions, clamping and cutting the umbilical cord, completing administrative procedures, and performing household tasks. Focusing on these tasks afforded them a sense of control.

Getting things under control, for example, having the car ready, it is important not to run out of gas. To know who the other daughter is going to stay with and, if she has to go to school, who is going to take her and pick her up (13:9).

Previous experience was recognized as a facilitating aspect because it increased awareness and situated partners more actively within the process. This familiarity enabled partners to approach subsequent births with greater confidence and emotional preparedness.

This second time, I went through it more consciously, more naturally. When things happen to us several times, we begin to normalise them, and the truth is that I went through it positively (8:3).

#### 3.2.2 Subtheme 2: Barriers to the Development of the Caregiver Role

The following factors hinder the performance of the caregiver role: caregiver needs, exclusion from the process, feelings of being lost, and undervaluation of care.

Participants conceptualized the family unit as a trio (woman–partner–newborn), rather than a dyad (woman–newborn), which reflected their desire to participate in caregiving. To maintain their capacity to care for the woman and the newborn, participants requested that their basic needs, such as food, hygiene, and rest be addressed, particularly while in the postnatal ward.

In the postpartum period, the role of the partner is fundamental, but at the same time we don't matter much. We can't use the bathroom; if it's the weekend, in the afternoons and evenings the cafeteria is closed; sometimes, you went to buy a hot sandwich, and the machine didn't work. You feel like a bit of a stowaway (29:34).

Partners were occasionally excluded from antenatal education classes, admission to the emergency department and from procedures such as epidural catheter placement. Such exclusion frequently provoked frustration and helplessness, since partners expressed a strong desire to be actively involved throughout the care continuum.

Her trusted person, her support, the one who is watching everything from the beginning, has always been myself. <No, you wait outside> but let me explain what's going on, or how things went. <You, get out> (3:3).

It is very important to explain why this professional (anaesthesiologist) did not pay much attention to me. <Get out, please> if I had not had prior information, someone else would have stood up (7:9).

From the very beginning, at the healthcare centre they told us that partners could not attend (20:26).

Lack of information, receipt of contradictory information and being a first‐time parent made partners feel lost, generating fear and insecurity.

My wife had an intrapartum fever, and the baby had tachycardia. A lot of people began to come in; no one told us anything. I didn't know what was happening; there was a lack of information. Not knowing what is going on creates more anguish (1:13).

I think it is essential that they give you support and guide you, especially if you are a first‐timer. You have no idea how that's going to go. They have explained it to you a million times, but you have to live through it, you have to be there and experience it. What they tell you or what they put in a book is not the same as what you later endure, your own experience (1:24).

During the postpartum period, there also has to be a unification of criteria. At that moment, you don't know how to respond (14:63).

Finally, during childbirth and the postpartum period, partners tended to undervalue the care they provided. However, they acknowledged the same actions more positively when performed by a healthcare professional.

My role is basically like that of the midwife, who was right next to me and seemed to do nothing but give her confidence (3:42).

At first, it seemed to me that I was like a spare part, because I could do nothing but hold her hand or encourage her and little else (10:13).

I am aware of what my role is: to help my wife in any way I can, to be close to her and to be there, to be aware of some things that need to be done because I have a cooler head than the mother does and that is my role. I can't do much more (26:25).

**FIGURE 2:**
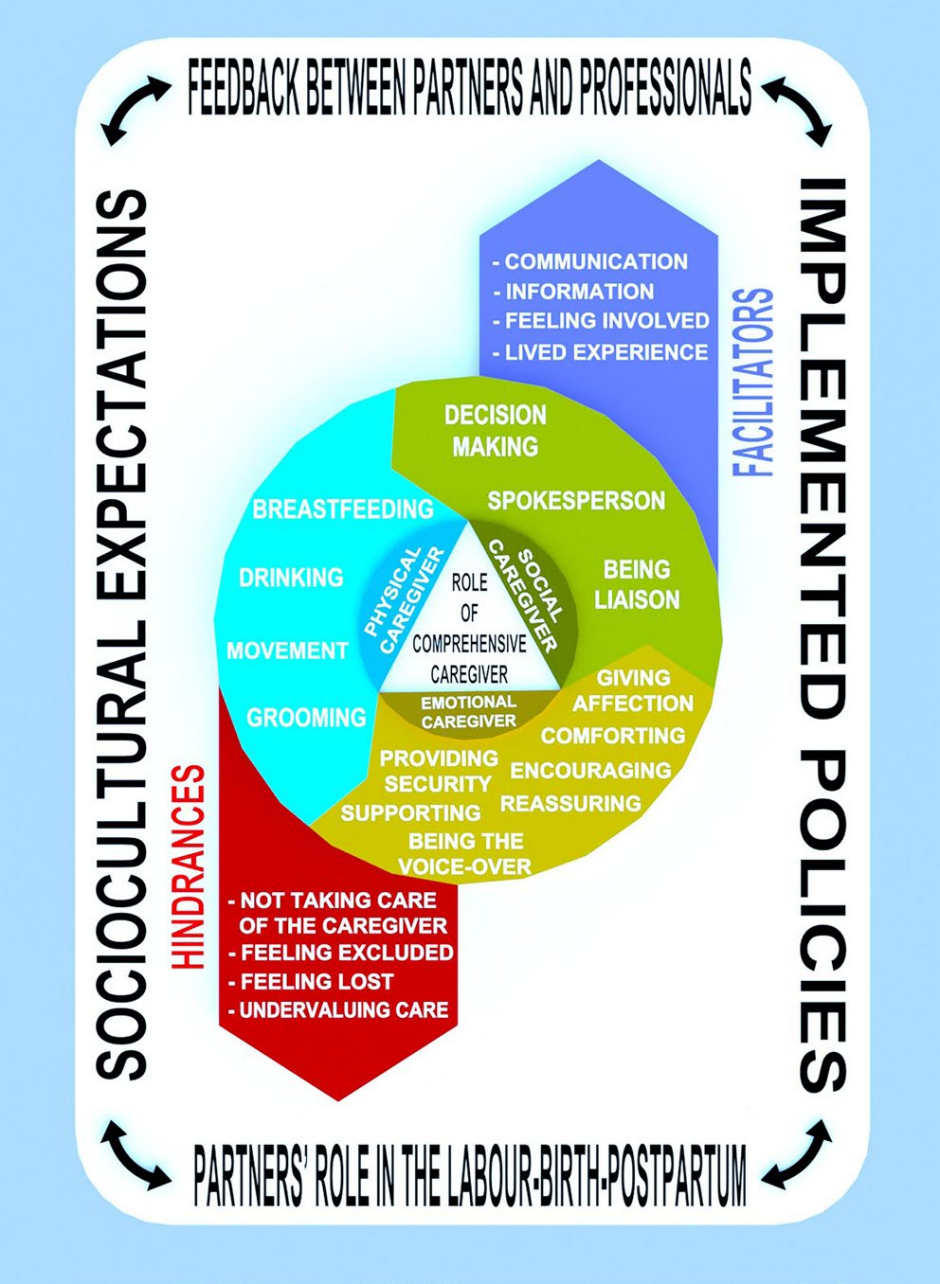
Role of comprehensive caregiver for partners during labor, birth, and the postpartum period.

Figure 2 provides a visual summary that integrates the elements addressed in this study. At the centre of the figure is the role of the comprehensive caregiver, which comprises three aspects, physical, emotional, and social care that encompass the actions described in the results. This role is influenced by facilitating and hindering factors, represented in the figure by upward and downward arrows, respectively. Moreover, three conditioning factors influence these factors: sociocultural expectations, current policies, and feedback between partners and healthcare professionals.

## 4 Discussion

The results of this study provide an updated perspective on the roles that partners play during labor, birth, and the postpartum period, highlighting an evolution relative to previous research. In their ethnographic study, Longworth et al. (2021) classified partners' roles during labor and birth as active or passive, consistent with Chapman (1992) earlier typology. However, in our study, all partner roles were active.

Longworth et al. (2021) further concluded that partners adopted four roles during childbirth: observer, caregiver, intermediary, and advocate, in response to contextual influences that serve the overarching goal of “protecting” the woman. In our study, a single multidimensional role was identified, that of caregiver, which included three aspects: providing physical, emotional, and social care.

Harrison et al. (2024) described partners as performing specific practical tasks, and Wanyenze et al. (2022) reported that partners provide emotional and physical support, but did not emphasize the social dimension highlighted in our study. In this context, social care encompassed acting as spokesperson, intermediary, and decision‐maker. The intermediary role (exchange of information) and the advocate role (communicating prior decisions) identified by Longworth et al. (2021) corresponded in the present study to the roles of liaison and spokesperson, respectively. Regarding the liaison function, in addition to serving as a link between professionals and external contacts, we identified a novel nuance: acting as the partner's link to the woman when the newborn required admission.

In relation to decision‐making, this study provided a novel perspective that expanded existing knowledge. Whereas previous studies (Shareef et al. 2024; Vahtel et al. 2021) examined this issue in isolation from the partner's role, the present study findings demonstrated that decision‐making was embedded within the partner's social role, manifesting in three forms: consensual, supportive, and autonomous.

The findings of the study make it possible to link the participation of partners in childbirth with Jean Watson's Theory of Human Caring (Watson 2008), by conceptualizing care as a transpersonal relationship that promoted well‐being from a holistic perspective. In this context, partners assumed a multidimensional role by providing physical, emotional, and social support, and by recognizing the woman as a whole being. This involvement not only strengthened the bond but also transformed the childbirth experience into a shared and meaningful process. Watson (2008) argued that caring had a transformative effect on both the caregiver and the recipient, constituting a co‐created process in which both were mutually acknowledged. Thus, partners become comprehensive caregivers, actively involved in creating an environment of trust, respect, and companionship.

These findings not only support Watson's conceptualization of caring as a transpersonal, holistic process, but also extend existing theoretical perspectives by integrating multiple dimensions of the partner's role—physical, emotional, and social—into a single comprehensive caregiver role. This challenges previous classifications of partner involvement as strictly active or passive (Longworth et al. 2021), highlighting the transformative potential of partner participation in perinatal care and its broader implications for family‐centred care models.

With respect to the factors that facilitated the performance of the caregiver role, the results obtained in this study aligned with those of previous studies. Communication with professionals was highlighted by Harrison et al. (2024); access to information by Griffith et al. (2025); Nambile Cumber et al. (2024); the feeling of being a participant by Schmitt et al. (2022); and previous lived experience by Mulugeta et al. (2024). In relation to information, it is noteworthy that, despite its recognized importance, more than half of the partners did not attend the education classes prior to childbirth. In this regard, the implementation of policies at different organizational levels to promote attendance seems appropriate (Leavy‐Warren et al. 2022).

With respect to difficulties in performing the caregiver role, the results of this study identified two factors not previously addressed in the literature: inadequate care of the caregiver and undervaluation of their role. Regarding the first factor, and in contrast to Harrison et al. (2024) and Uribe‐Torres et al. (2024), who concluded that partners ignored their own needs to prioritize the woman's needs, this study's findings indicated that partners sought care for themselves, particularly during the postpartum period. Addressing this difficulty and ensuring care for the caregiver requires a broad, multi‐level approach. Allport et al. (2018) reported that socioeconomic, geographical and social factors, including gender bias and restrictive gender norms, act as barriers to fathers' inclusion in family healthcare services at macro (societal and healthcare policy), meso (organizational policy) and micro (clinical practice) levels. Similarly, Watkins et al. (2024) identified barriers related to individual and organizational factors and persistent gender norms, noting that health services often remain primarily focused on women's needs. Likewise, Mwakyusa et al. (2025) and Smith et al. (2024) highlighted the need to promote policies that ensure more equitable and inclusive perinatal care. Regarding the second factor, the undervaluation of partners' role may reflect the continued invisibility of care tasks, which lack appropriate recognition (Hooyman 2024).

In summary, this research provided an updated perspective on the role of partners during the perinatal process, addressing a gap in the literature by including experiences from diverse family configurations. It also identified new barriers warranting consideration in the design of professional interventions.

These findings support conceptualizing the comprehensive caregiver role as a framework for developing training programmes that promote the recognition and active valuation of partners' participation in perinatal care. Inclusion of this perspective in professional education could facilitate more equitable, sensitive, and family‐centred care. However, several limitations should be considered.

### 4.1 Strengths and Limitations

This study is distinguished by its inclusive approach, considering diverse family configurations, including a same sex couple, which modestly broadened participant representativeness. Even though the findings are situated within a specific cultural and temporal context, as is typical in qualitative research, the aim is not statistical generalizability, but rather to provide an in‐depth understanding that may be transferable to similar contexts. Additionally, the sample was limited to vaginal deliveries, as institutional policy restricts partner presence during caesarean sections. Future research should independently explore partners' roles in caesarean births to provide a more comprehensive understanding of their involvement across different delivery modes.

## 5 Conclusions

This study contributes to a clearer definition of partners' role during labor, birth, and postpartum, consolidating it as a single role of comprehensive caregiver for the woman and the newborn. This role encompasses three dimensions of care: physical, emotional, and social, with particular emphasis on the social dimension, especially decision‐making. It is a dynamic, continually evolving role influenced by sociocultural expectations, feedback between partners and professionals, and implemented policies. Furthermore, previously identified facilitating factors were reaffirmed, and two novel hindrances were recognized: inadequate care of the caregiver and undervaluation of the partner's role.

## 6 Relevance for Clinical Practice

These results provide valuable information for healthcare professionals to optimize person‐centred care and to promote family‐oriented care models. Professionals play a key role in the construction of the role of partners, either by guiding them, motivating them to actively participate, or respecting their desire not to do so, thus promoting their role as caregivers of the woman and the newborn. This comprehensive approach seeks to empower partners, foster positive experiences throughout the perinatal process, and ensure a secure start to family life.

To maximize practical impact, institutional policies and clinical practices should be designed to promote complete partner integration throughout the perinatal continuum. These may include partner‐inclusive antenatal education, structured opportunities for partner engagement during labor and delivery, and supportive postnatal programs that acknowledge and sustain the partner's caregiving role. Implementing these practices can help to provide a safe and nurturing start to family life.

## Author Contributions

All of the authors contributed intellectually to the work, meet the conditions of authorship and have approved the final version of it. Study design: B.P.‐G. and M.U.‐I. Data collection: B.P.‐G., M.F.‐S., S.T.‐R. Data analysis: B.P.‐G., M.F.‐S., S.T.‐R. and J.X.H.‐E. Manuscript writing: B.P.‐G., M.F.‐S., S.T.‐R. and J.X.H.‐E. Work on the advanced version of the manuscript: B.P.‐G., M.F.‐S., S.T.‐R., M.U.‐I. and J.X.H.‐E. Final approval of the version to be published: B.P.‐G, M.F.‐S., S.T.‐R., M.U.‐I. and J.X.H.‐E. I declare that the work is original, has not been previously published and is not being reviewed by any other journal. Ethical principles have been respected at all times when carrying out the research, and we would be willing to provide more information about our data and methods if necessary.

## Funding

The research protocol of this project received the Mustela Award for Midwifery Research from the Spanish Federation of Midwifery Associations (Federación Española de Asociaciones de Matronas—FAME) and the Mustela Foundation in 2022 (11/2022). The founder of the prize was not involved in the conceptualisation of the study, data collection and analysis, publication decision, or manuscript preparation. Open Access funding was provided by the University of the Basque Country (12/2025).

## Ethics Statement

The Clinical Research Ethics Committee of the Health Area of Gipuzkoa approved this study (reference: BPG‐PHP‐2022‐01). The participants received oral and written information about the purpose of the research and signed the informed consent. The anonymity of the participants was guaranteed through the use of codes. Paper data were kept under lock and key, and digital information was held in a protected account. After the completion of the study, all the participants received feedback on the results.

## Conflicts of Interest

The authors declare no conflicts of interest.

## Data Availability

The data that support the findings of this study are available from the corresponding author upon reasonable request.

## References

[nhs70280-bib-0001] Allport, B. S. , S. Johnson , A. Aqil , et al. 2018. “Promoting Father Involvement for Child and Family Health.” Academic Pediatrics 18: 746–753. 10.1016/j.acap.2018.03.011. 29653255

[nhs70280-bib-0002] Baldwin, S. , M. Malone , J. Sandall , and D. Bick . 2018. “Mental Health and Wellbeing During the Transition to Fatherhood: A Systematic Review of First Time Fathers' Experiences.” JBI Database of Systematic Reviews and Implementation Reports 16: 2118–2191. 10.11124/JBISRIR-2017-003773. 30289768 PMC6259734

[nhs70280-bib-0003] Bohren, M. A. , G. J. Hofmeyr , C. Sakala , R. K. Fukuzawa , and A. Cuthbert . 2017. “Continuous Support for Women During Childbirth.” Cochrane Database of Systematic Reviews 7: CD003766. 10.1002/14651858.CD003766.pub6. 28681500 PMC6483123

[nhs70280-bib-0004] Chapman, L. L. 1992. “Expectant Fathers' Roles During Labor and Birth.” Journal of Obstetric, Gynecologic, and Neonatal Nursing 21: 114–120. 10.1111/j.1552-6909.1992.tb01729.x. 1607980

[nhs70280-bib-0005] Denvir, E. , S. W. Lindow , and M. P. O'Connell . 2025. “How Same Sex Couples Attending Maternity Services Would Like to Be Addressed.” European Journal of Obstetrics, Gynecology, and Reproductive Biology 307: 279–282. 10.1016/j.ejogrb.2025.02.013. 39952811

[nhs70280-bib-0006] Dubey, K. , N. Sharma , D. Chawla , R. Khatuja , and S. Jain . 2023. “Impact of Birth Companionship on Maternal and Fetal Outcomes in Primigravida Women in a Government Tertiary Care Center.” Cureus 15: e38497. 10.7759/cureus.38497. 37273329 PMC10237517

[nhs70280-bib-0007] Eatough, V. , and J. Smith . 2017. “Interpretative Phenomenological Analysis.” In The SAGE Handbook of Qualitative Research in Psychology, 193–211. SAGE Publications Ltd. 10.4135/9781526405555.n12.

[nhs70280-bib-0008] Elmir, R. , and V. Schmied . 2022. “A Qualitative Study of the Impact of Adverse Birth Experiences on Fathers.” Women and Birth 35: e41–e48. 10.1016/j.wombi.2021.01.005. 33495131

[nhs70280-bib-0009] Eustat . 2025. “Live Births by Place of Birth in the Basque Country by Province and Nationality of the Mother 2024.” https://en.eustat.eus/elementos/ele0014500/ti_nacimientos‐vivos‐por‐lugar‐del‐parto‐en‐la‐ca‐de‐euskadi‐territorio‐historico‐y‐nacionalidad‐de‐la‐madre‐2024/tbl0014581_i.html.

[nhs70280-bib-0010] Fisher, S. D. , J. Cobo , B. Figueiredo , et al. 2021. “Expanding the International Conversation With Fathers' Mental Health: Toward an Era of Inclusion in Perinatal Research and Practice.” Archives of Women's Mental Health 24: 841–848. 10.1007/s00737-021-01171-y. 34431009

[nhs70280-bib-0011] Friese, S. 2017. “ATLAS.Ti 8 Windows (Version 8.4) [Qualitative Data Analysis Software].” ATLAS.ti Scientific Software Development GmbH.

[nhs70280-bib-0012] Griffith, D. M. , E. C. Jaeger , P. Pepperman , K. A. Chustz , D. Frazier , and A. Wilson . 2025. “Expectant and New Fathers Say They Need Resources and Sources of Support.” BMC Pregnancy and Childbirth 25: 205. 10.1186/s12884-025-07290-z. 40011810 PMC11863926

[nhs70280-bib-0013] Harrison, G. , K. Fitzgerald , P. O'Leary , A. Kothari , and L. Callaway . 2024. “Promoting Men‐Inclusive Maternity Services: Exploring the Expectations, Experiences and Needs of Men as Fathers.” BMC Pregnancy and Childbirth 24: 1–9. 10.1186/s12884-024-06644-3. 38997650 PMC11245863

[nhs70280-bib-0014] Hodgson, S. , J. Painter , and L. Kilby . 2021. “The Experiences of First‐Time Fathers in Perinatal Services: Present but Invisible.” Healthcare (Basel) 9: 161. 10.3390/healthcare9020161. 33546202 PMC7913323

[nhs70280-bib-0015] Hoffmann, L. , N. Hilger , E. Riolino , A. Lenz , and R. Banse . 2023. “Partner Support and Relationship Quality as Potential Resources for Childbirth and the Transition to Parenthood.” BMC Pregnancy and Childbirth 23, no. 1: 435. 10.1186/s12884-023-05748-6. 37312055 PMC10261844

[nhs70280-bib-0016] Hooyman, N. 2024. Care Justice: Reframing Public Policy, Elevating Care Work. 1st ed. Routledge.

[nhs70280-bib-0017] Kabakian‐Khasholian, T. , and A. Portela . 2017. “Companion of Choice at Birth: Factors Affecting Implementation.” BMC Pregnancy and Childbirth 17, no. 1: 265. 10.1186/s12884-017-1447-9. 28854903 PMC5577840

[nhs70280-bib-0018] Leavy‐Warren, P. , L. Philpott , R. Elmir , and V. Schmied . 2022. “Fathers' Perceptions and Experiences of Support to Be a Parenting Partner During the Perinatal Period: A Scoping Review.” Journal of Clinical Nursing 32: 3378–3396. 10.1111/jocn.16460. 35898120

[nhs70280-bib-0019] Lincoln, Y. S. , and E. G. Guba . 1985. Naturalistic Inquiry. Sage Publications.

[nhs70280-bib-0020] Longworth, M. K. , C. Furber , and S. Kirk . 2021. “Fathers' Roles Matter Too: An Ethnographic Study Examining Fathers' Roles and the Influences on Their Roles During Labour and Birth.” Midwifery 92: 102857. 10.1016/j.midw.2020.102857. 33186868

[nhs70280-bib-0021] Michałek‐Kwiecień, J. , M. Kaźmierczak , and K. Karasiewicz . 2022. “Closeness With a Partner and Parental Bond With a Child During the Transition to Parenthood.” Midwifery 105: 103209. 10.1016/j.midw.2021.103209. 34890879

[nhs70280-bib-0022] Mulugeta, C. , T. Emagneneh , G. Kumie , A. Sisay , and A. Alamrew . 2024. “Male Partner Involvement in Delivery Care Service and Associated Factors in Ethiopia : A Systematic Review and Meta ‐ Analysis.” BMC Health Services Research 24: 1467. 10.1186/s12913-024-11993-y. 39587565 PMC11590235

[nhs70280-bib-0023] Mwakyusa, M. O. , A. Said , S. Selemani , et al. 2025. “If My Husband Was in the Labor Ward With Me, My Baby Wouldn't Have Died; Experiences on Birth Companionship From a Tertiary Health Facility, Tanzania.” PLoS One 20, no. 1: e0309602. 10.1371/journal.pone.0309602. 39787258 PMC11717287

[nhs70280-bib-0024] Nambile Cumber, S. , A. Williams , H. Elden , and M. Bogren . 2024. “Fathers' Involvement in Pregnancy and Childbirth in Africa: An Integrative Systematic Review.” Global Health Action 17: 2372906. 10.1080/16549716.2024.2372906. 38993149 PMC11249146

[nhs70280-bib-0025] Ngai, F. W. , and X. Xiao . 2021. “Perceptions of Paternal Involvement and Labour Pain Management in Chinese Couples During Childbirth: A Qualitative Study.” Women and Birth 34: 288–295. 10.1016/j.wombi.2020.03.003. 32222355

[nhs70280-bib-0026] Osakidetza – Basque Health Service . 2018a. “Atención Al Parto de Bajo Riesgo en El Medio Hospitalario [Low‐Risk Childbirth Care in the Hospital Setting] (Institutional Guideline).” Administration of the Autonomous Community of the Basque Country. https://www.osakidetza.euskadi.eus.

[nhs70280-bib-0027] Osakidetza – Basque Health Service . 2018b. “Atención al Puerperio [Postpartum Care] (Institutional Guideline).” Administration of the Autonomous Community of the Basque Country. https://www.osakidetza.euskadi.eus.

[nhs70280-bib-0028] Palioura, Z. , A. Sarantaki , E. Antoniou , M. Iliadou , and M. Dagla . 2023. “Fathers' Educational Needs Assessment in Relation to Their Participation in Perinatal Care: A Systematic Review.” Healthcare (Basel) 11: 200. 10.3390/healthcare11020200. 36673568 PMC9859150

[nhs70280-bib-0029] Roberts, J. , and H. Spiby . 2020. “‘The Calm Before the Storm’: A Qualitative Study of Fathers' Experiences of Early Labour.” Women and Birth 33: 490–495. 10.1016/j.wombi.2019.11.002. 31771817

[nhs70280-bib-0030] Saunders, B. , J. Sim , T. Kingstone , et al. 2018. “Saturation in Qualitative Research: Exploring Its Conceptualization and Operationalization.” Quality and Quantity 52: 1893–1907. 10.1007/s11135-017-0574-8. 29937585 PMC5993836

[nhs70280-bib-0031] Schmitt, N. , S. Striebich , G. Meyer , A. Berg , and G. M. Ayerle . 2022. “The Partner's Experiences of Childbirth in Countries With a Highly Developed Clinical Setting: A Scoping Review.” BMC Pregnancy and Childbirth 22, no. 1: 742. 10.1186/s12884-022-05014-1. 36192684 PMC9528111

[nhs70280-bib-0032] Shareef, N. , P. Said , S. Lamers , M. Nieuwenhuijze , M. de Vries , and J. van Dillen . 2024. “The Contribution of Birth Plans to Shared Decision‐Making From the Perspectives of Women, Their Partners and Their Healthcare Providers.” PLoS One 19: e0305226. 10.1371/journal.pone.0305226. 38924004 PMC11207161

[nhs70280-bib-0033] Small, A. , S. A. Kavanagh , J. A. Macdonald , L. Di Manno , and K. Wynter . 2025. “Father Involvement in Pregnancy and Postnatal Care: Combined Perspectives of Fathers, Mothers, and Service Providers.” Nursing and Health Sciences 27: e70105. 10.1111/nhs.70105. 40254548 PMC12009787

[nhs70280-bib-0034] Smith, C. , C. Pitter , and D. A. Udoudo . 2024. “Fathers' Experiences During Delivery of Their Newborns: A Content Analysis.” International Journal of Community Based Nursing and Midwifery 12: 23–31. 10.30476/ijcbnm.2023.100009.2337. 38328009 PMC10844877

[nhs70280-bib-0035] Smith, J. , P. Flowers , and M. Larkin . 2022. Interpretative Phenomenological Analysis. 2nd ed. SAGE Publications Ltd.

[nhs70280-bib-0036] Sun, Y. , P. L. Chui , M. C. Chong , J. Zhang , R. H. Guo , and F. Xu . 2025. “Fathers' Emotional Experiences and Cultural Perspectives During Childbirth: A Qualitative Meta‐Synthesis.” Nursing and Health Sciences 27: e70185. 10.1111/nhs.70185. 40673574

[nhs70280-bib-0037] Tong, A. , P. Sainsbury , and J. Craig . 2007. “Consolidated Criteria for Reporting Qualitative Research (COREQ): A 32‐Item Checklist for Interviews and Focus Groups.” International Journal for Quality in Health Care 19: 349–357. 10.1093/intqhc/mzm042. 17872937

[nhs70280-bib-0038] Uribe‐Torres, C. , M. M. Serrano , P. B. Valenzuela , M. B. Silva , J. Bilardi , and M. Temple‐Smith . 2024. “Paternal Well‐Being Perception During Childbirth: Experience of Prepared Chilean Fathers After a Prenatal Education Intervention.” Revista da Escola de Enfermagem da USP 58: e20240009. 10.1590/1980-220X-REEUSP-2024-0009en. PMC11533973 39475390

[nhs70280-bib-0039] Vahtel, K. , K. Eilmann , J. Pühvel , and M. Kangasniemi . 2021. “Expectant Fathers' Experiences of Family‐Centred Births in Estonia: A Qualitative Study.” Midwifery 96: 102948. 10.1016/j.midw.2021.102948. 33631412

[nhs70280-bib-0040] Vischer, L. C. , X. Heun , J. Steetskamp , A. Hasenburg , and C. Skala . 2020. “Birth Experience From the Perspective of the Fathers.” Archives of Gynecology and Obstetrics 302: 1297–1303. 10.1007/s00404-020-05714-z. 32740868 PMC7524830

[nhs70280-bib-0041] Wanyenze, E. W. , J. K. Byamugisha , N. M. Tumwesigye , P. A. Muwanguzi , and G. K. Nalwadda . 2022. “A Qualitative Exploratory Interview Study on Birth Companion Support Actions for Women During Childbirth.” BMC Pregnancy and Childbirth 22, no. 1: 63. 10.1186/s12884-022-04398-4. 35073861 PMC8785438

[nhs70280-bib-0042] Watkins, V. , S. A. Kavanagh , J. A. Macdonald , et al. 2024. “‘I Always Felt Like I Wasn't Supposed to be There’. An International Qualitative Study of Fathers' Engagement in Family Healthcare During Transition to Fatherhood.” Midwifery 130: 103928. 10.1016/j.midw.2024.103928. 38290320

[nhs70280-bib-0043] Watson, J. 2008. Nursing: The Philosophy and Science of Caring. Rev. ed. University Press of Colorado.

[nhs70280-bib-0044] World Health Organization . 2020a. “WHO, 2020. Every Woman's Right to a Companion of Choice During Childbirth.” [WWW Document]. https://www.who.int/news/item/09‐09‐2020‐every‐woman‐s‐right‐to‐a‐companion‐of‐choice‐during‐childbirth.

[nhs70280-bib-0045] World Health Organization . 2020b. “Companion of Choice During Labour and Childbirth for Improved Quality of Care.” Publ. World Heal. Organ. 1–7. https://www.who.int/publications/i/item/WHO‐SRH‐20.13.

[nhs70280-bib-0046] Wynter, K. , L. Di Manno , V. Watkins , B. Rasmussen , and J. A. Macdonald . 2021. “Midwives' Experiences of Father Participation in Maternity Care at a Large Metropolitan Health Service in Australia.” Midwifery 101: 103046. 10.1016/j.midw.2021.103046. 34098224

